# Fungal endophyte *Phomopsis liquidambari* affects nitrogen transformation processes and related microorganisms in the rice rhizosphere

**DOI:** 10.3389/fmicb.2015.00982

**Published:** 2015-09-17

**Authors:** Bo Yang, Xiao-Mi Wang, Hai-Yan Ma, Teng Yang, Yong Jia, Jun Zhou, Chuan-Chao Dai

**Affiliations:** ^1^Jiangsu Key Laboratory for Microbes and Functional Genomics, Jiangsu Engineering and Technology Research Center for Industrialization of Microbial Resources, College of Life Sciences, Nanjing Normal University, NanjingChina; ^2^State Key Laboratory of Soil and Sustainable Agriculture, Institute of Soil Science, Chinese Academy of Sciences, NanjingChina

**Keywords:** fungal endophyte, ammonia-oxidizing archaea (AOA), ammonia-oxidizing bacteria (AOB), diazotroph, rhizosphere, root exudate

## Abstract

The endophytic fungus *Phomopsis liquidambari* performs an important ecosystem service by assisting its host with acquiring soil nitrogen (N), but little is known regarding how this fungus influences soil N nutrient properties and microbial communities. In this study, we investigated the impact of *P. liquidambari* on N dynamics, the abundance and composition of N cycling genes in rhizosphere soil treated with three levels of N (urea). Ammonia-oxidizing archaea (AOA), ammonia-oxidizing bacteria (AOB) and diazotrophs were assayed using quantitative real-time polymerase chain reaction and denaturing gradient gel electrophoresis at four rice growing stages (S0: before planting, S1: tillering stage, S2: grain filling stage, and S3: ripening stage). A significant increase in the available nitrate and ammonium contents was found in the rhizosphere soil of endophyte-infected rice under low N conditions. Moreover, *P. liquidambari* significantly increased the potential nitrification rates, affected the abundance and community structure of AOA, AOB, and diazotrophs under low N conditions in the S1 and S2 stages. The root exudates were determined due to their important role in rhizosphere interactions. *P. liquidambari* colonization altered the exudation of organic compounds by rice roots and *P. liquidambari* increased the concentration of soluble saccharides, total free amino acids and organic acids in root exudates. Plant-soil feedback mechanisms may be mediated by the rice-endophyte interaction, especially in nutrient-limited soil.

## Introduction

Biotic processes, such as symbioses, can improve agricultural sustainability with less reliance on non-renewable inputs such as synthetic fertilizers (Cardoso and Kuyper, 2006). For example, numerous studies suggest that symbionts can enhance growth and confer biotic and abiotic stress tolerance to host plants (Redman et al., 2002; Schardl et al., 2004). Fungal endophytes have been frequently reported to enhance host plant growth and also improve plant nutrient capture from soil (Lyons et al., 1990; Upson et al., 2009). Our previous research indicated that a fungal endophyte, *Phomopsis liquidambari*, can produce 3-indoleacetic acid and abscisic acid *in vitro* (Yuan et al., 2004; Chen et al., 2011). Cross-host species inoculation of rice with *P. liquidambari* revealed that this endophyte forms a mutualistic symbiotic relationship with rice (Yang et al., 2014a), promotes the growth and yield of rice (Yuan et al., 2007), improves the N accumulation and N use efficiency of rice (Yang et al., 2014b), and significantly reduces the required amount of soil N fertilizer (Li et al., 2009). Moreover, previous work reported that *P. liquidambari* stimulates the expression of several genes involved in N-uptake and the metabolism of rice seedlings (Yang et al., 2014a). These results indicate the beneficial effects of *P. liquidambari* on N use in rice plants, but the mechanisms involved are largely unknown.

Soil microbe communities are intrinsically linked to agroecosystem functioning through N-mineralization, nitrification, N-fixation, and consequently generate nitrate (NO_3_^-^) or ammonium (NH_4_^+^) for plant utilization and influence plant N-uptake and production (Wardle et al., 2004; Bardgett et al., 2005). Previous studies have demonstrated that plants are able to select for functional bacterial groups such as ammonium-oxidizers or N-fixers to create an optimal environment for their growth (Briones et al., 2003; Cocking, 2003). Soil ammonia-oxidizers and N-fixers are two important microbial groups participating in N transformation (Falkowski et al., 2008). Microbial ammonia oxidation is the first and rate-limiting step of nitrification, and this process is carried out by ammonia-oxidizing bacteria (AOB) and ammonia-oxidizing archaea (AOA) (Gruber and Galloway, 2008). Microbial N-fixation is an important source of N input in many natural ecosystems. The capacity for N-fixation is unique to certain groups of bacteria and archaea that contain the highly conserved gene *nifH*, which encodes the iron protein subunit of nitrogenase (Zehr et al., 2003; Raymond et al., 2004). Some studies have reported that arbuscular mycorrhizal (AM) fungi can influence the abundance and community of ammonia-oxidizers and potential nitrification rates (PNR) (Veresoglou et al., 2011; Chen et al., 2013d). In addition, colonization by fungal endophytes causes successional shifts in the rhizospheric or phyllospheric microbial communities (Casas et al., 2011; Yang et al., 2013). Our previous studies indicated that *P. liquidambari* exhibited efficient degradation of N-heterocyclic indole (Chen et al., 2013c), and addition of this fungus to soil effectively alters the balance of soil microbes in the rhizosphere and even promotes mineral N release via changing soil organic N distribution (Chen et al., 2013a,b). Thus, we hypothesized that *P. liquidambari in vivo* can affect the diversity and abundance of ammonia-oxidizers and N-fixers in the rice rhizosphere, increase available soil N, and improve N-uptake by rice.

Many different selection factors are likely to determine rhizosphere microbial abundance and composition. This includes the composition and quantity of root exudates and other carbon substrates provided by rhizodeposition of sloughed off cells and root debris, which varies depending on rhizosphere microsite location and plant species (Grayston et al., 1998; Marschner et al., 2001). Soil microbes are often limited by energy in soils, and the rhizosphere of rice is highly dynamic as soil micro-biota respond rapidly to changes in chemical composition and quantities of root exudates (Whipps, 2001; Rengel, 2002; Lu et al., 2006). Soluble saccharides, free amino acids, and organic acids are the three main types of carbon and nitrogen sources in plant rhizospheric exudates and may stimulate the growth of microbial populations capable of influencing the biogeochemical cycling of C, N, S, and P (Fontaine and Barot, 2005; Bais et al., 2006). Therefore, our second hypothesis was that infection by *P. liquidambari* alters rice root exudates of organic compounds.

## Materials and Methods

### Fungal Strain

The endophytic fungus *P. liquidambari* was isolated from the inner bark of *Bischofia polycarpa* (Shi et al., 2004). The fungus was stored at 4°C on potato dextrose agar (PDA, containing 200 g L^-1^ potato extract, 20 g L^-1^ glucose, and 20 g L^-1^ agar, pH 7.0).

### Plant Seeds and Paddy Soils

The rice cultivar used was a japonica subspecies of *Oryza sativa* L. “Wuyunjing 7” (a common cultivar grown in the Jiangsu Province of south-eastern China). The rice seeds were gently dehulled, soaked in 96% ethanol for 15 min, rinsed twice with sterile distilled water (SDW), sterilized in 0.1% HgCl_2_ for 25 min in 0.1% HgCl_2_, and rinsed six times in SDW (Chaintreuil et al., 2000). The sterilized seeds and the water from the last washing were placed on PDA plates at 28°C for 5 days as a sterility check (Feng et al., 2006).

Paddy soils were collected from the experimental rice fields of Nanjing Normal University, Jiangsu Province, China (32° 6.318′ N, 118° 54.88′ E). The soil for the experiment (yellow–brown loam) was air-dried, mixed to homogeneity, sieved (2-mm mesh) to remove plant tissues, weighed, and then added to plastic pots (25 cm diameter, 35 cm high). The organic matter content was 1.08%. The soil pH was 5.97. The nutrient composition of the soil was: available N, 98.64 mg kg^-1^; total N, 1.21 g kg^-1^; available P, 24.67 mg kg^-1^; total P, 0.39 g kg^-1^; available K, 67.58 mg kg^-1^; and total K, 0.73 g kg^-1^.

### Endophytic Fungus Inoculation and Cultivation of Rice Seedlings

*Phomopsis liquidambari* was cultured in 100 mL of potato dextrose broth (PDB, pH 5.5) in Erlenmeyer flasks (250 mL) for 3 days at 28°C at 160 rpm and then submerged fermentation was performed in 1-L flasks containing 500 mL of a sterilized PDB medium and 10% seed culture broth for 4 days (at 28°C at 160 rpm). 10 mL of culture broth was then used to determine the dry cell weight by washing the fungal mycelia with SDW twice and drying the mycelia at 80°C in an oven to a constant dry weight. Totally, 3.03 g (up to 0.33 g dry weight) of fungal mycelia was harvested, washed twice with SDW, and then diluted with SDW to a final volume of 200 mL. The fungal suspension was used as the agent on the germinating seeds.

The sterilized seeds were divided into two groups randomly and then transferred to Petri dishes (20 cm diam., 100 grains per dish). The inoculated group (E+) was treated with 80 mL of the fungal agent described above for each dish. For the non-inoculated group (E-), 80 mL of sterilized deionized water was used as a control (Tian et al., 2007). The seeds were germinated and grown in Petri dishes for 6 days in a growth cabinet (29/25°C day/night, with a light intensity of 250 μmol m^-2^ s^-1^ and a photoperiod of 16 h). Germinated rice seeds were transplanted into pots containing 15 kg of paddy soil for plant growth. Seedlings at similar developmental stages were transplanted into pots (1 seedling per hill, 3 hills per pot, 15 kg soil per pot) after 30 days of growth (mid-June 2013) and grown in the experimental rice field conditions. This region has a typical subtropical monsoon climate with a mean annual temperature of 15.8°C and an annual precipitation of 1107 mm. Before transplanting, some of the rice seedlings were randomly sampled and processed for microscopy to examine *P. liquidambari* infection (Supplementary Figure S1).

### Experimental Design and Plant Growth Conditions

The experimental design was the same as reported in the previous study (Yang et al., 2014b). Plants were arranged in a 3 × 2 factorial design, in which the main effects were the endophyte and the amount of N applied. N treatments consisted of three gradients: 1.25 g of N per pot (low N), 2.5 g of N per pot (medium N), and 3.75 g of N per pot (high N). N was applied as urea in three separate applications: (1) 7 days before planting (around 60% of total N), (2) at the tillering stage (30% of total N), and (3) at the panicle differentiation stage (10% of total N). Before planting, potassium (1.4 g of K_2_O per pot) and phosphorus (1.6 g of P_2_O_5_ per pot) were applied as basal dressing. Plants were regularly watered during the growing season.

### Sample Collection and Preparation

Soil samples were collected at different rice growing stages: before planting (flooded condition; S0), 45 days after planting (tillering stage, 7 days after topdressing, flooded condition; (S1), 81 days after planting [grain filling stage, wet condition; (S2)] and 110 days after planting (ripening stage, moist condition; (S3). Before planting (S0), soil samples were collected from three treatments (LN, MN, and HN). Six replicate pots were used for each treatment (S0). After planting, 10 replicate pots were grown and maintained for each treatment (LN, MN, and HN; E+ and E-; three plants per pot). Rhizosphere soil was operationally defined as soil adhering to the total roots after gentle shaking. The whole plants with their roots were excavated to a 15-cm depth from soil, and at each growth stage, after shaking off the loosely adhering soil, the tightly adhering soil (rhizosphere soil) was carefully collected. To obtain enough rhizosphere soil for assays, at each growth stage (S1, S2, and S3), six plants were randomly selected from three pots in each treatment (two plants per pot), and the rhizosphere soils from the same pot were pooled to form one composite sample. Soil samples were sieved (2 mm mesh) immediately after collection, and aliquots for DNA extraction were stored at -80°C. The remaining soil used for chemical analysis was stored at 4°C. The sampling depth was the same throughout the rice growing stages for comparative purposes. All soil samples were taken at same time (9:30 AM) on each sampling day in order to eliminate diurnal effects.

### Chemical Analysis and PNR

Vario MACRO Cube Element Analyzer (Elementar Analysensysteme GmbH, Hanau, Germany) was used to measure total N content. The NH_4_^+^ and NO_3_^-^ contents were measured by extracting the soil with 0.01 mol L^-1^ CaCl_2_ solution (1:10, w/v) for 30 min and then determining with a flow injection autoanalyzer (FLA star 5000 Analyzer, Foss, Denmark). A chlorate inhibition method was used to determine the PNR according to Kurola et al. (2005). Briefly, 5.0 g of fresh soil was placed in 50-mL centrifuge tubes containing 20 mL of phosphate buffer solution (PBS, NaH_2_PO_4_, 0.2 g L^-1^; Na_2_HPO_4_, 0.2 g L^-1^; KCl, 0.2 g L^-1^; NaCl, 8.0 g L^-1^; pH 7.4) with 1 mmol L^-1^ (NH_4_)_2_SO_4_. Potassium chlorate with a final concentration of 10 mmol L^-1^ was added to inhibit nitrite oxidation. The suspension was incubated in an incubator at 25°C for 24 h at dark, and nitrite was extracted with 5 mL of 2 mol L^-1^ KCl and determined by a spectrophotometer at 540 nm with N-(1-naphthyl) ethylenediamine dihydrochloride.

### DNA Extraction and Real Time PCR Assay

The soil DNA was extracted from 0.5 g of soil using the UltraClean Soil DNA isolation Kit (MoBio Loker Ave West, Carlsbad, CA, USA). Extracts were characterized by electrophoresis on 1.5% agarose gels, and serial dilutions were used to assess whether a PCR inhibition by impurities of the DNA extracts. The *amoA* gene (encodes the alpha subunit of AMO) and *nifH* gene (encodes the iron protein subunit of nitrogenase) have been widely used as functional gene markers to analyze the phylogeny and abundance of AOB, AOA, and diazotroph in natural environments (Zehr et al., 2003; Raymond et al., 2004; Bürgmann et al., 2005; Francis et al., 2005; Wartiainen et al., 2008). The copy numbers of *Arch-amoA* (AOA), *amoA* (AOB) and *nifH* genes in all soil samples were determined in triplicate using an ABI 7500 Real-Time PCR System (Applied Biosystems). The thermocycling conditions were as follows: 30 s at 95°C, followed by 40 cycles of 5 s at 95°C, 60 s at 58°C for AOA, 45 s at 60°C for AOB, and 35 s at 60°C for diazotroph, and 60 s at 72°C. Fluorescence was read during each cycle at 83°C. The primers are listed in **Table 1** without GC clamps. The DNA concentration of all soil samples was measured at 260 nm by using a UV Spectrophotometer (Bio Photometer, Eppendorf, Germany) and then adjusted to 10 ng μL^-1^. Each reaction was performed in a 25-μL volume containing 10 ng of DNA, 0.2 mg mL^-1^ BSA, 0.2 μmol L^-1^ of each primer and 12.5 μL of SYBR premix EX Taq TM (Takara, Shinga, Japan). The amplification specificity of the PCR products was confirmed by electrophoresis analysis on 1.5% agarose gel and generating a melting curve.

**Table 1:** Primers used for the real-time PCR and PCR-DGGE analyses.

Target group	Name	Sequence (5′–3′)	Length of amplicon (bp)	Reference
Ammonia-oxidizing bacteria (AOB; *amoA* gene)	amoA1F	GGGGTTTCTACTGGTGGT	490	Rotthauwe et al., 1997
	GC^a^-amoA1F			
	amoA2R	CCCCTCKGSAAAGCCTTCTTC		
Ammonia-oxidizing archaea (Arch-*amoA* gene)	Arch-amoAF	STAATGGTCTGGCTTAGACG	630	Francis et al., 2005
	Arch-amoAR	GCGGCCATCCATCTGTATGT		
	GC^b^-Arch-amoAR			
Diazotroph (*nifH* gene)	Pol F1	TGC GAI CCS AAI GCI GAC TC	300	Wartiainen et al., 2008
	AQER	GAC GAT GTA GAT YTC CTG		
	GC^c^-AQER			

*S = C/G; K = G/T; Modified bases; I = Inosine*

*^a^(CGC CCG CCG CGC CCC GCG CCC GTC CCG CCG CCC CCG CCC G)*

*^b^(CGC CCG CCG CGC CCC GCG CCC GGC CCG CCG CCC CCG CCC C)*

*^c^(CGC CCG CCG CGC CCC GCG CCC GGC CCG CCC).*

A plasmid standard containing the target region was generated for each primer set (diazotroph, AOA and AOB) using the total DNA extracted from the soil samples. The amplified PCR products of the *nifH, Arch-amoA* (AOA), and *amoA* (AOB) genes were purified using a PCR solution purification kit (Takara), ligated into a p-GEM T easy vector (Promega, Madison, WI, USA) and cloned into *Escherichia coli* DH5α. Clones containing correct inserts were selected as the standards for real-time PCR (qPCR). Plasmid DNA concentrations were measured by spectrophotometry as mentioned above. Standard curves were generated using triplicate 10-fold dilutions of plasmid DNA ranging from 1.14 × 10^2^ to 1.14 × 10^8^ copies for *Arch-amoA* (AOA) gene, 1.05 × 10^2^ to 1.05 × 10^8^ copies for *amoA* (AOB) gene and 2.19 × 10^2^ to 2.19 × 10^8^ copies of template for *nifH* gene per assay. High amplification efficiencies of 97.8% (AOA), 98.4% (AOB), and 99.7% (*nifH*) were obtained using the slopes -3.37, -3.36, and -3.33 of the standard curve, as calculated by the formula Eff = [10^(-1/slope)^-1] × 100%, respectively. The copy numbers of *nifH* and *amoA* genes (AOA and AOB) gene were calculated based on standard curves.

### PCR Amplification and DGGE Analysis of the Community of Ammonia-Oxidizers and Diazotrophs

PCRs were performed using an Eppendorf C1000 Touch Thermal Cycler (Eppendorf, Hamburg, Germany). *amoA* for AOB, *Arch-amoA* for AOA and *nifH* for diazotroph were amplified with the primer pairs containing a GC-clamp in one primer (**Table 1**). The 50-μL reaction mixtures consisted of 5 μL of 10 × PCR buffer (Mg^2+^ free), 4 μL of 2.5 mmol L^-1^ dNTPs, 3 μL of 25 mmol L^-1^ MgCl_2_, 1 μL of each primer at 50 mmol L^-1^, 0.5 μL of Taq polymerase (5 U μL^-1^, Takara), and 1 μL of DNA template (10–15 ng). To amplify bacterial and archaeal *amo*A gene fragments, the touchdown PCR strategy used included 3 min at 94°C, and a touchdown procedure (94°C for 40 s, annealing for 45 s at temperatures decreasing from 60 to 55°C during the first 10 cycles, and ending with an extension step at 72°C for 1 min). This was followed by 30 additional cycles of 94°C for 40 s, 55°C for 45 s, 72°C for 1 min, and, finally, an extension at 72°C for 10 min. For the *nifH* gene, the PCR strategy was initial denaturation at 95°C for 3 min, followed by 35 cycles of 94°C for 1 min, 55°C for 1 min and 72°C for 1 min, with a final extension at 72°C for 10 min.

To separate the partially amplified *amoA*, *Arch-amoA*, and *nifH* genes of different sequences, DGGE analyses were performed with the DCode Universal Mutation Detection System (Bio-Rad Laboratories, Hercules, CA, USA) as follows. The PCR amplicons of bacterial and archaeal *amo*A genes were separated on a 6% (*w/v*) acrylamide–bisacrylamide gel with a 40–70% and 20–55% denaturing gradient (the 100% denaturant contained 7 mol L^-1^ urea and 40% formamide), respectively. The *nifH* gene amplicon was separated on an 8% (*w/v*) acrylamide–bisacrylamide gel, using denaturing gradients of 40–70%. In order to make the DGGE analysis results comparable, we expected to run the DNA samples of all treatments at the same stage in the one DGGE gel-sheet. However, due to that there were six treatments at each stage, one gel-sheet could not afford to run all the three replicate DNA extracts for each treatment. Additionally, our pretest experiments found that the consistency between the three replicates in each treatment was relatively high (data not shown); and two replicates of three could cover and represent almost all the bands in the same treatment. Therefore, for DGGE analyses, two replicates were randomly chosen for each treatment. Similarly, every replicate represented all mixed genetic information from one pot. After running for 16 h at 100 V, the gels were stained with SYBR Green I (1:10000, Invitrogen Molecular Probes, Eugene, OR, USA) for 30 min and then scanned by a GelDOC-ItTS imaging system (Ultra Violet Products, Upland, CA, USA).

### Collection and Analysis of Root Exudates

In a separate experiment, germinated rice seeds (both E- and E+ groups were obtained as mentioned previously) were washed and transferred to buckets containing 2 L of sterile nutrient solution for solution culture. The components of nutrient solution were added according to the International Rice Research Institute (Yoshida et al., 1976) with some modifications. There were three levels of N treatments: 1.0 mmol L^-1^ N (low N), 3.0 mmol L^-1^ N (medium N), and 5.0 mmol L^-1^ N (high N), with NH_4_NO_3_ used as the N source. The nutrient solution contained 1.6 mmol L^-1^ MgSO_4_, 1.0 mmol L^-1^ CaCl_2_, 0.6 mmol L^-1^ K_2_SO_4_, 0.32 mmol L^-1^ NaH_2_PO_4_, 0.2 mmol L^-1^ Na_2_SiO_3_, 0.072 mmol L^-1^ Fe-EDTA, 18.5 μmol L^-1^ H_3_BO_3_, 9.1 μmol L^-1^ MnCl_2_, 0.154 μmol L^-1^ ZnSO_4_, 0.156 μmol L^-1^ CuSO_4_, and 0.526 μmol L^-1^ H_2_MoO_4_, with the pH adjusted to 5.2. The nutrient solution was replaced daily. The buckets were maintained in a growth chamber (29/25°C day/night, with a light intensity of 250 μmol m^-2^ s^-1^ and a photoperiod of 16 h).

After 14 days of growth, 10 seedlings were transplanted in a glass tube containing 50 mL of nutrient solution (three N levels were mentioned previously) under sterile conditions. The seedlings were growth on wire gauze aseptically submerged in the nutrient solution in such a manner that grains rested on the gauze, roots dipped in nutrient solution and shoots in air. After 14 days of growth in a water bath in the phytotron with black ink to keep roots in the dark, the plants were harvested, and the medium contained the exudates was sterile filtered. Both the E- and E+ groups for each N treatment were replicated six times, and the exudates containing plant media from the same treatments were combined. A volume of 50 mL of exudates was evaporated using a rotary evaporator (Yarong Model RE-52A, Nanjing, China) at 40°C until reaching a volume of 5 ml. The exudates were kept at -80°C prior to analysis.

Soluble saccharides were detected by the anthrone colorimetry method (Yemm and Willis, 1954). The determination of organic acids was conducted by improving the method of Cawthray (2003). The separation and quantification of organic acids was accomplished using Agilent 1100 RPLC workstations (Agilent Technologies, USA) and an Agilent HC-C18 (2), analytical 4.6 mm × 250 mm 5-micro-type column. The mobile phase contained 93% 25 mmol L^-1^ KH_2_PO_4_ (adjusted to pH 2.4) and 7% methanol at a flow rate of 0.7 mL min^-1^. The column temperature of 25 ± 2°C and DAD output at 210 nm were proven to be effective and precise. An injection volume of 20 μL was applied in this method. The limit of detection (LOD) was defined as a ratio of 3 for signal-to-noise (S/N), and all values reported for LOD are based on peak area. Standard compounds were chromatographed alone and in mixture, and organic acids was identified by comparing standard retention times.

The root exudates for free amino acid analysis were pre-column derivatized with phenylisothiocyanate (PITC; Pierce). The PicoTag method (Waters, USA) was used as described by Cohen et al. (1989). Physiological amino acid standard solutions (acid/neutral and basics from Sigma) and a glutamine solution were used and external standards were prepared along with the samples. Norleucine was used as an internal standard. The standards and samples were analyzed using High-Performance Liquid Chromatography (HPLC) in a Waters Reversed-Phase Amino Acid Analysis System equipped with a PicoTag column (3.9 mm × 300 mm). The conditions were described by Cohen et al. (1989). The resulting peaks were analyzed with Breeze software (Waters).

### Statistical Analysis

The results of the DGGE profiles were analyzed using Gelcompar II (Applied Maths, Austin, TX, USA) software with default values. Gelcompar alignment and statistical analyses were only performed on individual gels. Cluster analysis of microbial profiles was performed using Gelcompar II to construct a dendrogram using unweighted pair group method (UPGMA) based on Pearson’s similarity coefficient calculated from the complete densitometric curves. Canonical correspondence analysis (CCA) was performed by vegan package (Oksanen et al., 2012) in R environment to show a visual relationship between environmental factors and N-related microbial distributions using relative band peak area intensity data from the Gelcompar II results. In addition, we tested for significant differences in community composition among different treatments (E- vs. E+ regardless of the N level) using analysis of similarities (ANOSIM) with R statistical software (R Development Core Team, 2010). Other basic statistical analyses were performed by SPSS 15.0 using a two-way ANOVA, followed by Duncan’s test to determine significant differences between the relative datasets. The results were considered as significant at *P* < 0.05. For some variables, a natural log transformation was used to obtain a normal distribution of the residuals and to homogenize the variance. Pearson’s correlation analysis was performed to assess the relationships between PNR and the abundance of AOA and AOB.

## Results

### Soil Chemical Properties and Potential Nitrification Rates

In general, both N treatment and *P. liquidambari* infection led to differences in soil N composition (**Table 2**). N addition significantly increased both NH_4_^+^ and NO_3_^-^ concentrations in rhizosphere soil (Supplementary Table S3). The NH_4_^+^ concentrations were significantly increased by *P. liquidambari* infection in rhizosphere soil under the low N condition and maintained concentrations 21.2–28.8% higher than that of the control, during the S1–S2 stages. The NH_4_^+^ concentrations of E+ treatments under medium N conditions were also 9.7% higher than the E- treatments at the S1 stage. Similarly, soil NO_3_^-^ concentrations were also increased by *P. liquidambari* infection in rhizosphere soil, being 41.1–41.9% higher in the E+ treatments than in E- treatments under low N conditions during the S1–S3 stages and 17.3–19.3% higher in the E+ treatments than in E- treatments under medium N conditions during the S1–S2 stages. The total N concentrations were not affected by *P. liquidambari*, but the proportion of NO_3_^-^-N to total inorganic N (NO_3_^-^-N + NH_4_^+^-N) was significantly increased by *P. liquidambari* infection under low N conditions, during the S1–S3 stages.

**Table 2:** Soil nutrient concentrations in the rhizospheric soil at four rice growing stages.

		Total N (g kg^-1^)		NH_4_^+^ (mg kg^-1^)		NO_3_^-^ (mg kg^-1^)		[NO_3_^-^/(NH_4_^+^ + NO_3_^-^)] (%)	
		E-	E+	E-	E+	E-	E+	E-	E+
S0	LN	1.25 ± 0.04		7.70 ± 0.27		0.79 ± 0.05		9.31 ± 0.53	
	MN	1.29 ± 0.02		10.85 ± 0.36		1.41 ± 0.08		11.50 ± 0.36	
	HN	1.34 ± 0.03		13.56 ± 0.41		3.02 ± 0.10		18.26 ± 0.33	
S1	LN	1.25 ± 0.04	1.22 ± 0.02	6.55 ± 0.21	7.94 ± 0.33**	1.12 ± 0.09	1.58 ± 0.13**	14.60 ± 0.26	16.60 ± 0.28**
	MN	1.28 ± 0.02	1.27 ± 0.01	9.73 ± 0.19	10.67 ± 0.45*	2.20 ± 0.15	2.58 ± 0.09**	18.44 ± 0.41	19.47 ± 0.52*
	HN	1.31 ± 0.03	1.32 ± 0.02	12.59 ± 0.30	13.05 ± 0.42	3.51 ± 0.16	3.39 ± 0.21	21.80 ± 0.25	20.62 ± 0.32
S2	LN	1.23 ± 0.02	1.27 ± 0.03	4.80 ± 0.25	6.18 ± 0.37**	0.80 ± 0.07	1.22 ± 0.16**	14.29 ± 0.39	16.49 ± 0.26**
	MN	1.28 ± 0.02	1.28 ± 0.02	6.93 ± 0.26	7.44 ± 0.21	1.66 ± 0.12	1.98 ± 0.08*	19.32 ± 0.65	21.02 ± 0.59*
	HN	1.30 ± 0.03	1.32 ± 0.03	10.05 ± 0.35	10.58 ± 0.27	2.47 ± 0.19	2.55 ± 0.15	19.73 ± 0.40	19.42 ± 0.53
S3	LN	1.19 ± 0.02	1.23 ± 0.05	3.42 ± 0.14	3.71 ± 0.22	0.43 ± 0.05	0.61 ± 0.13*	11.17 ± 0.33	14.12 ± 0.47*
	MN	1.28 ± 0.03	1.30 ± 0.04	5.34 ± 0.23	5.75 ± 0.26	1.07 ± 0.10	1.12 ± 0.08	16.69 ± 0.44	16.30 ± 0.41
	HN	1.31 ± 0.03	1.30 ± 0.05	7.97 ± 0.20	8.05 ± 0.25	1.97 ± 0.17	2.01 ± 0.11	19.98 ± 0.31	20.53 ± 0.39

Data are means ± SE from three biological replicates. * and ** indicate statistically significant differences between infected (E+) and uninfected (E-) plants (*P < 0.05, **P < 0.01). S0, unplanted soil; S1, tillering; S2, grainfilling; S3, ripening; LN, low N; MN, medium N; HN, high N.

**FIGURE 1:**
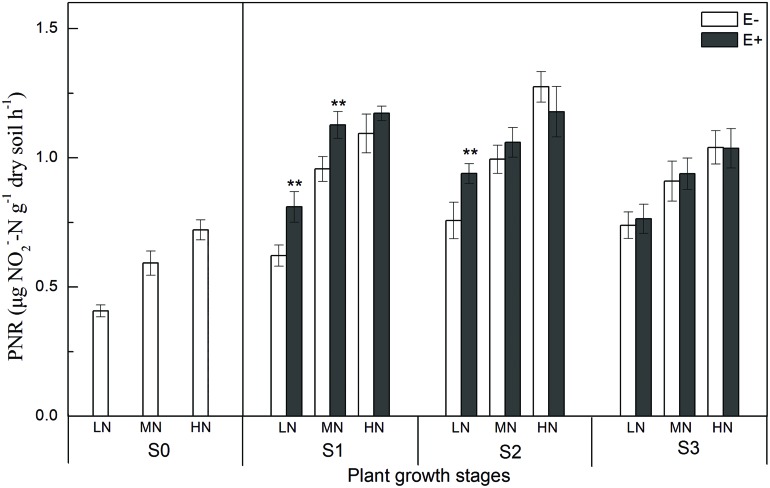
**Potential nitrification rates (PNRs) in rhizosphere soil at four rice growing stages (S0, unplanted soil; S1, tillering; S2, grainfilling; S3, ripening).** The values are the means ± SE from three biological replicates. ** indicates significant differences between E+ and E- plants (**P < 0.01). E+, endophyte infected; E-, endophyte uninfected; LN, low N; MN, medium N; HN, high N.

Potential nitrification rates, which provide an independent estimate of the abundance of ammonia-oxidizers, were significantly increased by increasing the available N concentration. Interestingly, the PNR of E+ treatments were 30.2% (*P* < 0.01) and 24.0% (*P* < 0.01) higher than that of E- treatments under low N conditions at the S1 and S2 stages (**Figure 1**). The growth stage of rice plants also influenced the PNR in rhizosphere soil, and the PNR increased soon after the rice transplanting with the maximum recorded at S2 stage.

### The Abundance of AOA, AOB, and Diazotrophs

**FIGURE 2:**
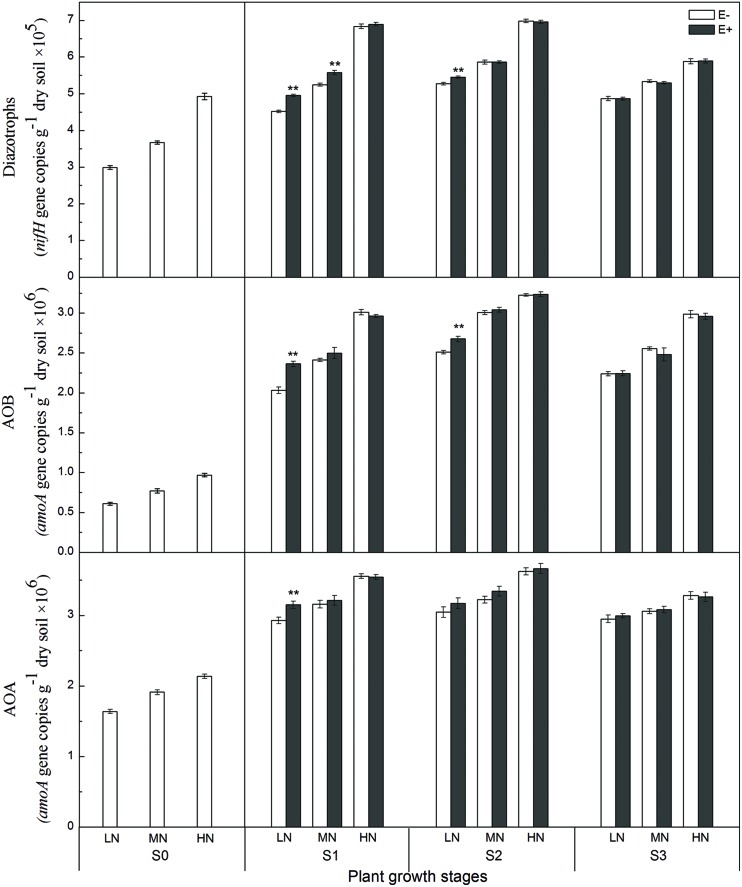
**Abundance of *amoA* [Ammonia-oxidizing archaea (AOA) and ammonia-oxidizing bacteria (AOB)] and *nifH* (diazotroph) genes in the rhizosphere soil at four rice growing stages (S0, unplanted soil; S1, tillering; S2, grainfilling; S3, ripening).** The values are the means ± SE from three biological replicates. ** indicates significant differences between E+ and E- plants (**P < 0.01). E+, endophyte infected; E-, endophyte uninfected; LN, low N; MN, medium N; HN, high N.

The abundance of AOA, AOB, and diazotrophs was determined by quantifying the copy numbers of *amoA* and *nifH* genes. The copy numbers of the archaeal *amoA* (AOA) gene in the paddy soil, ranging from 2.1 × 10^6^ to 4.9 × 10^6^ g^-1^ dry weight of soil, were greater than those of the bacterial *amoA* (AOB) gene, ranging from 0.9 × 10^6^ to 3.0 × 10^6^ g^-1^ dry weight of soil, during all growing stages. The copy numbers of the *nifH* gene ranged from 2.9 × 10^5^ to 7.0 × 10^5^ g^-1^ dry weight of soil, during all growing stages (**Figure 2**). Compared with the rhizosphere soil of E- plants, the AOA *amoA* gene abundances in E+ treatments were 8.2% (*P* < 0.01) higher under low N conditions at the S1 stage, and there was no apparent difference between the E- and E+ treatments at the other three stages. Similarly, the AOB *amoA* gene abundances in the E+ treatments were 17.3% (*P* < 0.01) and 7.0% (*P* < 0.01) higher than that in E- treatments under low N conditions at the S1 and S2 stages, respectively. The *nifH* gene abundances in E+ treatments were 9.7% and (*P* < 0.01) and 6.3% (*P* < 0.01) higher than that in E- treatments under low and medium N conditions at the S1 stage and 3.3% (*P* < 0.01) higher than that in E- treatments under low N conditions at the S2 stage.

N-fertilization levels were also found to significantly affect population sizes of AOA, AOB, and diazotrophs during all growing stages (Supplementary Table S4), and higher N fertilizer levels resulted in relatively higher *amoA* and *nifH* gene copy numbers. The growth stage of rice plants also influenced *amoA* and *nifH* gene abundances in rhizosphere soil, and the population sizes of AOA, AOB and diazotrophs increased soon after transplanting with the maximum abundances recorded at S2 stage. Moreover, the abundances of both AOB (*r* = 0.87, *P* < 0.01) and AOA (*r* = 0.86, *P* < 0.01) were significantly positive correlated with the PNR.

### Community Structure of AOA, AOB, and Diazotrophs

**FIGURE 3:**
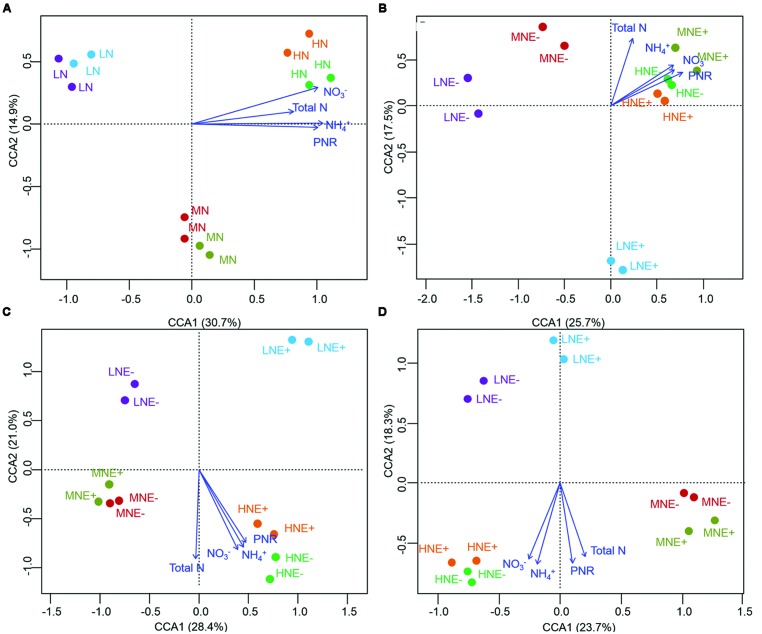
**Denaturing gradient gel electrophoresis (DGGE) profile and analysis of soil ammonium-oxidizing archaeal communities. (A–D)** Canonical correspondence analysis (CCA) of AOA communities generated by the AOA DGGE patterns of four different stages of rice **(A)** S0, unplanted soil; **(B)** S1, tillering; **(C)** S2, grainfilling; **(D)** S3, ripening. E+, endophyte infected; E-, endophyte uninfected; LN, low N; MN, medium N; HN, high N.

**FIGURE 4:**
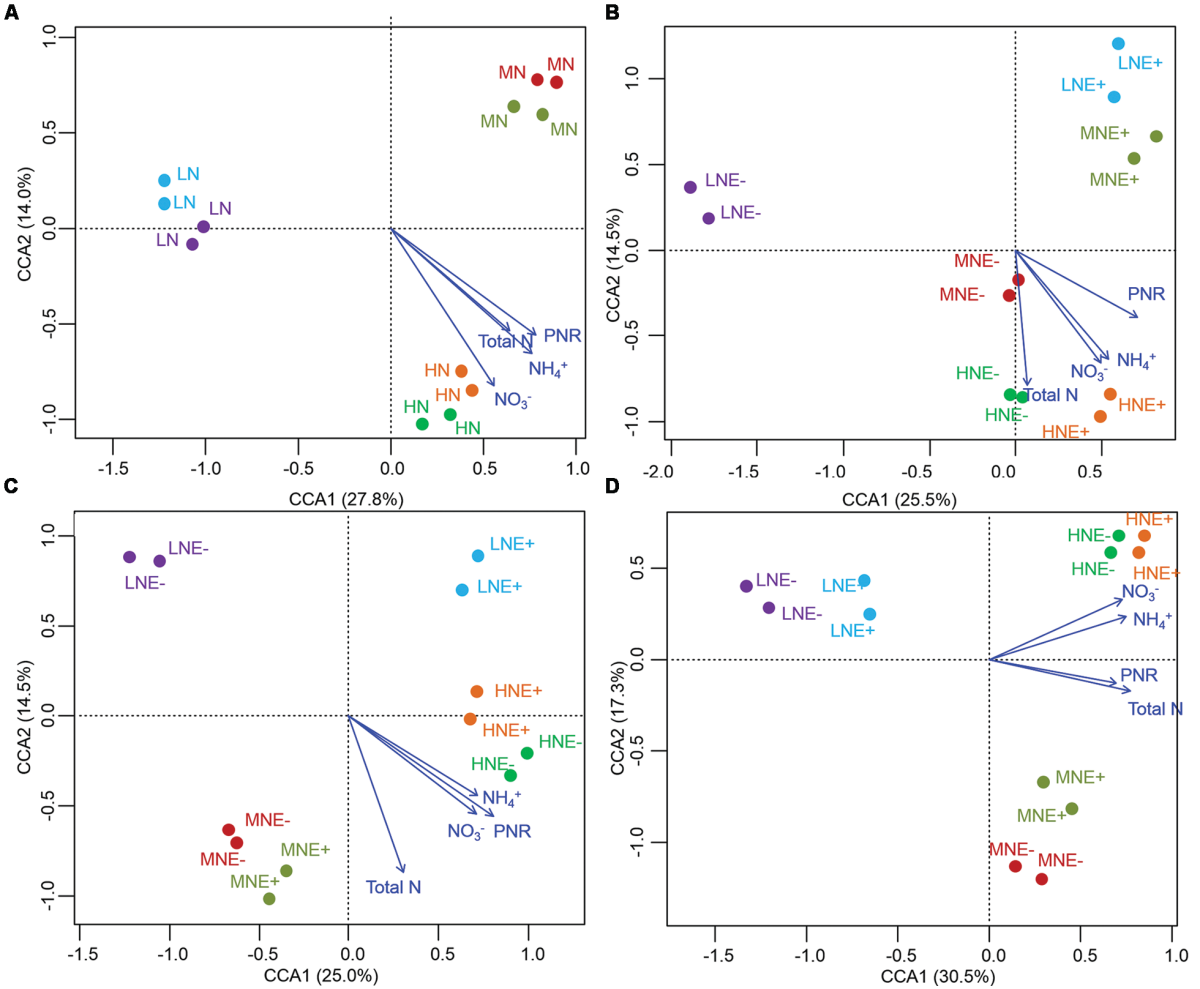
**Denaturing gradient gel electrophoresis profile and analysis of soil ammonium-oxidizing bacteria communities. (A–D)** CCA of AOB communities generated by the AOB DGGE patterns of four different stages of rice **(A)** S0, unplanted soil; **(B)** S1, tillering; **(C)** S2, grainfilling; **(D)** S3, ripening. E+, endophyte infected; E-, endophyte uninfected; LN, low N; MN, medium N; HN, high N.

**FIGURE 5:**
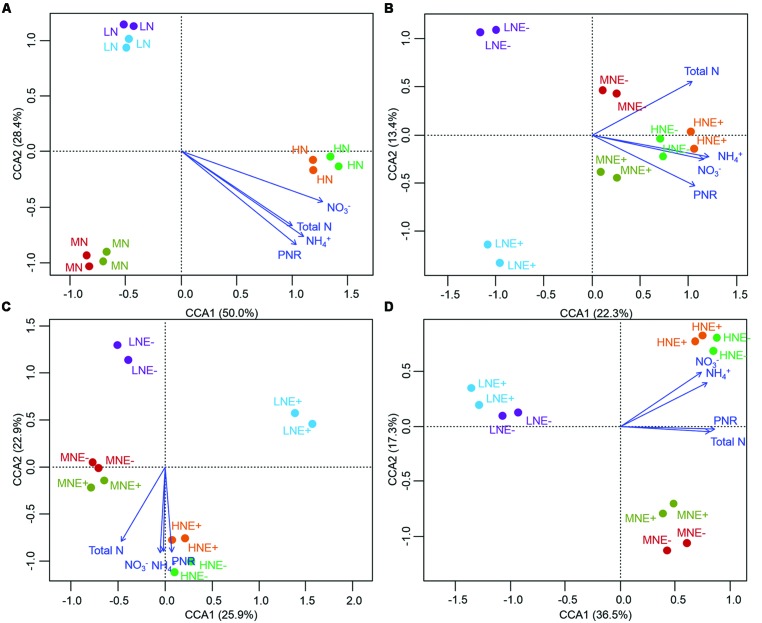
**Denaturing gradient gel electrophoresis profile and analysis of soil diazotroph communities. (A–D)** CCA of diazotroph communities generated by the diazotroph DGGE patterns of four different stages of rice **(A)** S0, unplanted soil; **(B)** S1, tillering; **(C)** S2, grainfilling; **(D)** S3, ripening. E+, endophyte infected; E-, endophyte uninfected; LN, low N; MN, medium N; HN, high N.

The community structure of the AOA, AOB, and diazotrophs in rhizosphere soils were characterized by PCR-DGGE in duplicate for all treatments. N-fertilization levels obviously altered the compositions of AOA, AOB, and diazotroph communities in some of the growing stages (**Figures 3–5**). Cluster analysis of PCR-DGGE band patterns of AOA *amoA* genes indicated that *P. liquidambari* symbiosis treatments (E+) showed low similarity with groups in the control (E-) treatments under low N conditions at both the S1 and S2 stages (less than 45% similarity), whereas high similarity was observed between E- and E+ treatments at other N levels during all stages (more than 60% similarity; Supplementary Figure S2). The CCA analysis also produced similar results, with the E+ treatments occupying an independent region separate from the E- treatments under low N conditions at both the S1 and S2 stages (**Figures 3A–D**). However, there was a relatively high similarity between the E+ and E- treatments under all three N conditions at the S3 stage, which might indicate that the effects caused by *P. liquidambari* did not lead to the AOA community being unrecoverable in the long term. Similarly, the community structures of the AOB and diazotrophs displayed similar temporal dynamics as AOA (**Figures 4** and **5**). There were no distinct differences between the E- and E+ treatments at all N levels during all stages except for under low N conditions at the S1 and S2 stages. The similarity between E+ and E- treatments under low N conditions at the S1 and S2 stages was less than 30 and 50% for AOB (Supplementary Figure S3) and was less than 40 and 15% for diazotrophs (Supplementary Figure S4), respectively. It is noteworthy that the obvious differences in the AOB and diazotroph community structure caused by *P. liquidambari* also nearly disappeared at the S3 stage (higher than 75% similarity), although different clusters were formed between the E+ and E- treatments under low N conditions. Moreover, CCA analysis showed that N contents (NH_4_^+^, NO_3_^-^, and total N) and PNR had strong effects on composition of AOA, AOB, and diazotrophs community (**Figures 3–5**). ANOSIM analysis showed that there were significant differences (*R* = 0.31, *P* = 0.043; *R* = 0.58, *P* = 0.004, respectively) of AOA and AOB communities between E- and E+ treatments at S1 stage, regardless of N levels. These results implied that the endophyte’s effects on the N-related microbial groups in rice rhizosphere were significant during S1 stage.

### Soluble Saccharides, Free Amino Acids and Low-Molecular-Weight Organic Acids in Root Exudates

Symbiosis with *P. liquidambari* obviously increased the concentration of soluble saccharides, total free amino acids and organic acids in rice root exudates (**Table 3**). Under low N conditions, the concentration of soluble saccharides in the E+ treatment (86.2 ± 4.0 μg mL^-1^) was 34.0% higher than in the E- treatment (64.3 ± 2.3 μg mL^-1^). The total free amino acid concentration in the E+ treatment was 79.5% higher than in the E- treatment. The total concentration of organic acids in the E+ treatment was 64.3% higher than in the E- treatment. The concentration of the individual amino acids and organic acids is listed in the Supplemental material (see Supplementary Tables S1 and S2).

**Table 3:** Concentration of soluble saccharides, free amino acids, and organic acids in root exudates.

	LN		MN		HN	
	E-	E+	E-	E+	E-	E+
Soluble sugar contents (μg mL^-1^)	64.35 ± 2.30	86.22 ± 4.01**	83.59 ± 5.56	96.22 ± 7.64	99.48 ± 8.93	110.44 ± 8.15
						
Organic acid contents (μg mL^-1^)	900.29 ± 67.76	1479.40 ± 109.31**	1650.77 ± 187.38	2040.80 ± 203.17	2234.26 ± 199.25	2532.04 ± 219.84
						
Free amino acid contents (μmol mL^-1^)	66.72 ± 12.08	119.74 ± 14.17**	100.94 ± 20.89	148.90 ± 23.56*	267.94 ± 24.84	298.38 ± 26.05

Data are means ± SE from three biological replicates. * and ** indicate statistically significant differences between infected (E+) and uninfected (E-) plants (*P < 0.05, **P < 0.01). LN, low N; MN, medium N; HN, high N.

## Discussion

Fungal endophytes are well known as symbionts of many plants, and they have received increasing attention because of their multiple functions. For example, they provide their host plants with mineral nutrients, protection against biotic (pathogens and herbivores) and abiotic (e.g., drought) stresses (Rodriguez et al., 2008; Upson et al., 2009). Previous studies have demonstrated that *P. liquidambari* has beneficial effects on N utilization in rice (Yang et al., 2014a,b), but the mechanisms are largely unknown. Our results indicated that the fungal endophyte has a role in improving the available N levels in rhizosphere soil of rice, shifting the rhizospheric microbial community involved in N turnover, and promoting rice N utilization (Yang et al., 2014b).

### Fungal Endophyte Shifts the Abundance and Community Structure of AOA, AOB, and Diazotrophs

Previous studies suggested that colonization by the fungal endophyte *Neotyphodium* in Italian rye grass caused changes in the bacterial community structure (Casas et al., 2011), and the endophyte presence can modify litter decomposition by changing the quality of the litter produced by infected plants and by altering micro-environmental conditions (Omacini et al., 2004; Lemons et al., 2005). Our results indicated that colonization by the fungal endophyte affects microbial abundance and composition (AOA, AOB, and diazotrophs) in rice rhizosphere soil. Both the PNR and the abundance of AOA, AOB, and diazotrophs were significantly increased by endophyte infection under low N conditions at the S1 and S2 stages. In contrast, Cavagnaro et al. (2007) observed no differences between mycorrhiza-defective mutant tomatoes and their wild-type progenitors in either the composition or the density of AOB. However, Veresoglou et al. (2011) demonstrated that mycorrhizal plants consistently supported lower PNR than non-mycorrhizal plants, and Chen et al. (2013d) reported that inoculation with AM fungi significantly decreased the abundance of AOA and AOB in the root compartment; their possible explanation was the AM fungi might occupy the same niche as ammonia-oxidizers and compete with them during NH_4_^+^ acquisition. Our previous studies also demonstrated that the endophytic fungus *P. liquidambari in vitro* stimulated organic mineralization and promoted NH_4_^+^-N release, and such effects triggered a soil AOB-driven nitrification process (Chen et al., 2013a).

It is noticeable that the changes in the abundance and composition of AOA, AOB, and diazotrophs caused by the fungal endophyte mainly occurred under low N conditions and gradually weakened with the increase of N-fertilization. These findings are in agreement with the report by Ravel et al. (1997) who found the beneficial effects of *Neotyphodium lolii* on the growth of perennial ryegrass mainly occurred under N deficiency. Similarly, Saari and Faeth (2012) found that the hybridization of symbiotic *Neotyphodium* increase the competitive potential of Arizona fescue in stressful environments and that this hybridization may be underlying niche expansion of the host in environments with low resources. By using quantitative PCR, our previous study estimated the endophyte densities in rice plants grown under different N levels and found that the endophyte’s density was significantly reduced in shoots and in roots with increased N levels (Yang et al., 2014a). Furthermore, a high N supply also reduced the fungal endophyte’s density in *Lolium perenne* (Rasmussen et al., 2007). In this study, given the effects of *P. liquidambari* on the contents of soil available N and the abundance and diversity of AOA, AOB, and diazotrophs mainly occurring under low N conditions, we suggest that the low-N fertilization might induce a physiological state of rice favorable for *P. liquidambari* symbiosis and that the resulting higher concentration of the fungus is necessary for the beneficial effects on plant performance. However, the endophyte’s effects on the N-related microbial composition in rice rhizosphere were only detected during early growth stage, and not as strong as the effect of N level. Thus, further studies will focus on the interactions between N-fertilization and the endophyte’s concentration in rice during all growing stages.

Ammonia oxidation was apparently the rate-limiting step in nitrification and was stimulated by N treatments (Ke et al., 2013). The availability of ammonia in natural environments could be a key factor in shaping the community structures of AOA and AOB (Alam et al., 2013). In our results, a higher N fertilizer level promoted the growth of ammonia-oxidizers and resulted in increasing trends of AOA and AOB abundance in paddy soil. Furthermore, the DGGE fingerprints of the *amoA* genes indicated that N fertilizer levels significantly altered the compositions of the AOA and AOB communities. Real-time PCR analysis demonstrated that archaeal ammonia-oxidizers were much more abundant than bacterial ammonia-oxidizers at all three N levels. These results agreed well with previous reports (He et al., 2007; Chen et al., 2008) and suggested that AOA may contribute significantly to the nitrification in the agricultural ecosystem than AOB. In addition, PNR was significantly correlated with the abundance of AOA (*r* = 0.86, *P* < 0.01) and AOB (*r* = 0.87, *P* < 0.01), which indicated that both AOA and AOB played an important role in ammonia oxidation in the soil. These results are consistent with previous reports by He et al. (2007) where both AOA and AOB played an important role in ammonia oxidation in upland red soil in China. However, some studies have reported that the net nitrification rate was only significantly correlated with the abundance of AOB (Di et al., 2009; Jia and Conrad, 2009) or AOA (Gubry-Rangin et al., 2010; Zhang et al., 2012). Such a discrepancy in the relative contributions of AOB and AOA may be due to various factors (Prosser and Nicol, 2008), such as soil type, N-fertilization and precipitation (Zhang et al., 2010; Chen et al., 2013e). Thus, it is necessary to further study the functions of AOB and AOA under a variety of conditions.

### Optimization of the Soil N Nutrition of Rice by Fungal Endophyte

N is consumed in the greatest quantities among the macronutrients required by plants, as it is required for the biosynthesis of amino acids and secondary metabolites. N availability, as NH_4_^+^or NO_3_^-^, varies between ecosystems, but it often limits plant growth (Escobar et al., 2006). In our results, analysis of the soil N composition indicated that the fungal endophyte significantly increased NH_4_^+^ and NO_3_^-^ concentrations in rice rhizosphere soil under low N conditions during the S1–S2 stages (**Table 2**), and this provides more available NH_4_^+^ and NO_3_^-^ for uptake by the rice plant. A possible explanation for this increased NH_4_^+^ and NO_3_^-^ concentrations in the rhizosphere soil of E+ plants could be the alteration of the abundance and community structure of diazotroph, AOA, and AOB by endophytic colonization. Ladha and Reddy (2003) reported that lowland rice can continuously give moderate but constant yields without N fertilizer for thousands of years. This sustainable farming system is supposed to be largely due to the N inputs from various biological nitrogen fixation (BNF) processes in flooded rice fields (Roger and Ladha, 1992; Boddey and Dobereiner, 1995). Zhu (1989) calculated that BNF contributed 16–21% of rice N, and Bei et al. (2013) reported that rice benefited greatly from biological N-fixation processes in flooded rice fields. In our experiment, more NH_4_^+^ would be fixed from N_2_ because of the higher copy number of *nifH* genes in E+ treatments. However, by using mRNA-based profiling of *nifH* genes, Knauth et al. (2005) found that not all diazotrophs in roots fix N at any time, and some of them may be highly active albeit not abundant. Therefore, mRNA-based studies are needed to identify active key N-cycling bacteria species in our experimental conditions, and to further explore the effects of the fungal endophyte on them.

On the other hand, NO_3_^-^-N is of exceptional importance for the growth of rice plants (Kronzucker et al., 1999, 2000). Kronzucker et al. (1999, 2000) found lowland rice was efficient in absorbing and assimilating NO_3_^-^ when compared with NH_4_^+^ and compared with other plant species. Many studies have indicated that the rice growth, yield, and N acquisition are enhanced significantly when both NH_4_^+^ and NO_3_^-^ sources are provided simultaneously, as compared with growth on NH_4_^+^ alone in solution culture (Youngdahl et al., 1982; Raman et al., 1995). NO_3_^-^ enhances NH_4_^+^ assimilation in some way, possibly through the NO_3_^-^-specific induction of additional pathways for NH_4_^+^ assimilation (Kronzucker et al., 1999; Britto and Kronzucker, 2004). Our results indicated that the fungal endophyte increased the proportion of NO_3_^-^ to total inorganic N, and this enhanced nitrate nutrition, which was beneficial for rice growth and N utilization. Moreover, urea-fertilized rice plants actually receive a mixture of NH_4_^+^ and NO_3_^-^ with ratios depending on the root-associated PNRs (Briones et al., 2003). Higher PNR, which were positively correlated with the abundance of AOA and AOB in our experiment, will lead to higher NO_3_^-^ concentrations in the rhizosphere available for root absorption and assimilation.

### The Possible Mechanisms of Microbial Community Changes Caused by the Fungal Endophyte

The results obtained from the quantitative real-time PCR and DGGE profile analyses indicated the rhizospheric microbial abundances and communities were affected by endophytes under low N conditions in most cases, but the underlying mechanism was not revealed. It is well known that the rhizosphere plays an active role in soil fertility and plant growth (Rovira, 1969). The quality and quantity of root exudates released into the soil might influence the structure and activity of soil microbial community and, in turn, plant growth, generating feedback loops (Wardle et al., 2004; Treonis et al., 2005). Organic substances released from rice roots serve as an important carbon and energy source for the microbial activities in the rhizosphere, and some of the organisms responsible for the turnover of C, N, S, and Fe (Kuzyakov, 2002; Lu et al., 2006). Moreover, oxygen is thought to leak from aerenchymatous tissue in rice, creating an oxic rhizosphere; the existence of oxic microhabitats provides a favorable environment for nitrification in paddy soils (Revsbech et al., 1999). Hence, oxygen and nutrition-releasing aerenchymatous rice plants may affect the composition of the ammonia-oxidizers and N-fixers’ communities in waterlogged paddy soil. Indeed, the population sizes of AOA, AOB, and diazotrophs in rhizosphere soil were significantly increased after plantation in our experiments.

Van Hecke et al. (2005) indicated that the presence of the symbiotic *N. coenophialum* might enhance rhizodeposition by tall fescue and consequently influence microbial mineralization processes in soil, and hosting a fungal endophyte has the potential to enhance plant nutrient supply indirectly via a stimulatory effect on the soil microbial biomass under nutrient limited conditions. Similarly, we cultured the E+ and E- plants and then collected their root exudates. The effects of *P. liquidambari* on the concentration of soluble saccharides, free amino acids, and low-molecular-weight organic acids in each treatment were assayed. Our results indicated that N fertilization alters the organic compounds of root exudates, and infection by *P. liquidambari* enhances root exudates of organic compounds by rice under low N conditions. Root exudates generally vary substantially between different plant species and genotypes and can depend on a variety of factors such as nutrient levels, disease, and stress (Lakshmanan et al., 2014). Our previous study found that the endophyte’s concentration was significantly reduced in rice with increased N levels (Yang et al., 2014a). We suggest that the low-N fertilization might alter the plant-endophyte physiological relationship and that the resulting higher concentration of *P. liquidambari* is necessary for the changes of root exudation. Additionally, the endophyte’s colonization within plants might affect the ‘priming effect’ of nutrition additions on N-transforming processes. Ma et al. (1999) and Nobili et al. (2001) demonstrated that the addition of negligible amounts of carbon could remove the C limitation of the microbial community, resulting in a disproportional increase in the rates of N mineralization and possible nitrification. Endophyte colonization of plants might result in large changes in the quantitative, qualitative, and spatial parameters of exudation, and an increase in root exudation to the surrounding soil could have a beneficial effect on N-transforming processes. Admittedly, the present data in our experiments cannot clearly confirm whether the endophyte-induced changes of root exudates directly influence the microflora in the rice rhizosphere, and thus, this hypothesis requires more integrated investigation in further studies. Moreover, some other undiscovered explanations for the observed effects beyond root exudates (e.g., fungal exudates, fungal effector, competitive antagonism, etc.) are also need to be explored in further study.

## Conclusion

Our data demonstrated that the fungal endophyte *P. liquidambari* enhances the contents of organic compounds in rice root exudates, changes the abundance and composition of ammonia-oxidizers and N-fixers in the rice rhizosphere under low N conditions, and induces a change in the content and ratio of inorganic N, although the endophyte’s effects on the N-related microbial composition were only detected during early growth stage, and not as strong as the effect of N level. These changes in functional gene distribution and N availability in the rhizosphere could have significant implications for soil productivity and plant N utilization, especially in N-limited soils. The present work, along with our previous researches, highlights the importance of the fungal endophyte for plant nutrient uptake in lower N input agriculture. Using this fungal agent will be almost certainly helpful for reducing fertilizers input and increasing plant productivity in sustainable agriculture.

## Conflict of Interest Statement

The authors declare that the research was conducted in the absence of any commercial or financial relationships that could be construed as a potential conflict of interest.
