# Supplementary material for: Fungal endophyte Phomopsis liquidambari affects nitrogen transformation processes and related microorganisms in the rice rhizosphere
Source: Front Microbiol. 2015 Sep 17;6:982. doi: 10.3389/fmicb.2015.00982 (PMC4585018; doi:10.3389/fmicb.2015.00982)
Supplement: Supplementary file 1 [file Data_Sheet_1.DOC]

***Supplementary Material***

**Fungal** **endophyte *Phomopsis liquidambari*** **affects nitrogen transformation processes and related microorganisms in the rice rhizosphere**

Bo Yang^a,b^, Xiao-Mi Wang^a,b^, Hai-Yan Ma^a^, Teng Yang^a,b^, Yong Jia^a,b^, Jun Zhou^a^, Chuan-Chao Dai^a,b*^

a. Jiangsu Key Laboratory for Microbes and Functional Genomics, Jiangsu Engineering and Technology Research Center for Industrialization of Microbial Resources, College of Life Sciences, Nanjing Normal University, Nanjing 210023, China

b. State Key Laboratory of Soil and Sustainable Agriculture, Institute of Soil Science, Chinese Academy of Sciences, Nanjing 210008, China

^*^Corresponding author: Tel: +86-025-85891382, Fax: +86-025-85891382， E-mail address: [daichuanchao@njnu.edu.cn](mailto:daichuanchao@njnu.edu.cn)

1. **Supplementary Figures**

**Fig. S1** Fluorescent microscopy analysis of fungal endophyte *Phomopsis liquidambari*-colonized rice seedlings. Rice seedlings were grown in the presence of GFP-tagged *P. liquidambari* for 5 weeks. Images were taken with a fluorescent microscope equipped with an argon laser. Excitation was at 488 nm. a, colony of GFP-tagged hyphae growing within the root cells (arrowheads), merged. Bars: 10 µm. b, GFP-tagged hyphae growing within the root cells (arrowheads), Bars: 50 µm.


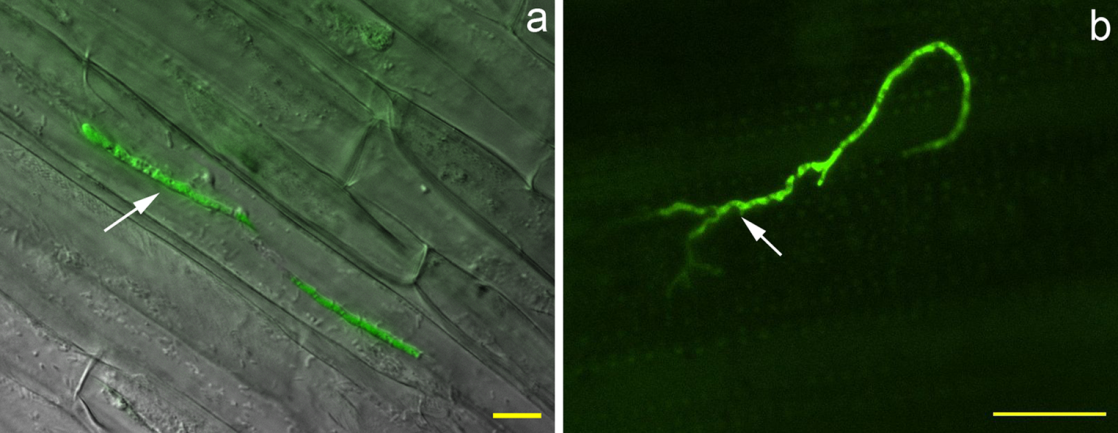


**Fig. S2** Denaturing gradient gel electrophoresis (DGGE) profile and analysis of soil ammonium-oxidizing archaeal communities. a-d: Cluster analysis of ammonium-oxidizing archaeal *amoA* genes from the DGGE pattern of four different stages of rice. (a: S0, unplanted soil; b: S1, tillering; c: S2, grainfilling; d: S3, ripening). E+, endophyte infected; E-, endophyte uninfected; LN, low N; MN, medium N; HN, high N.


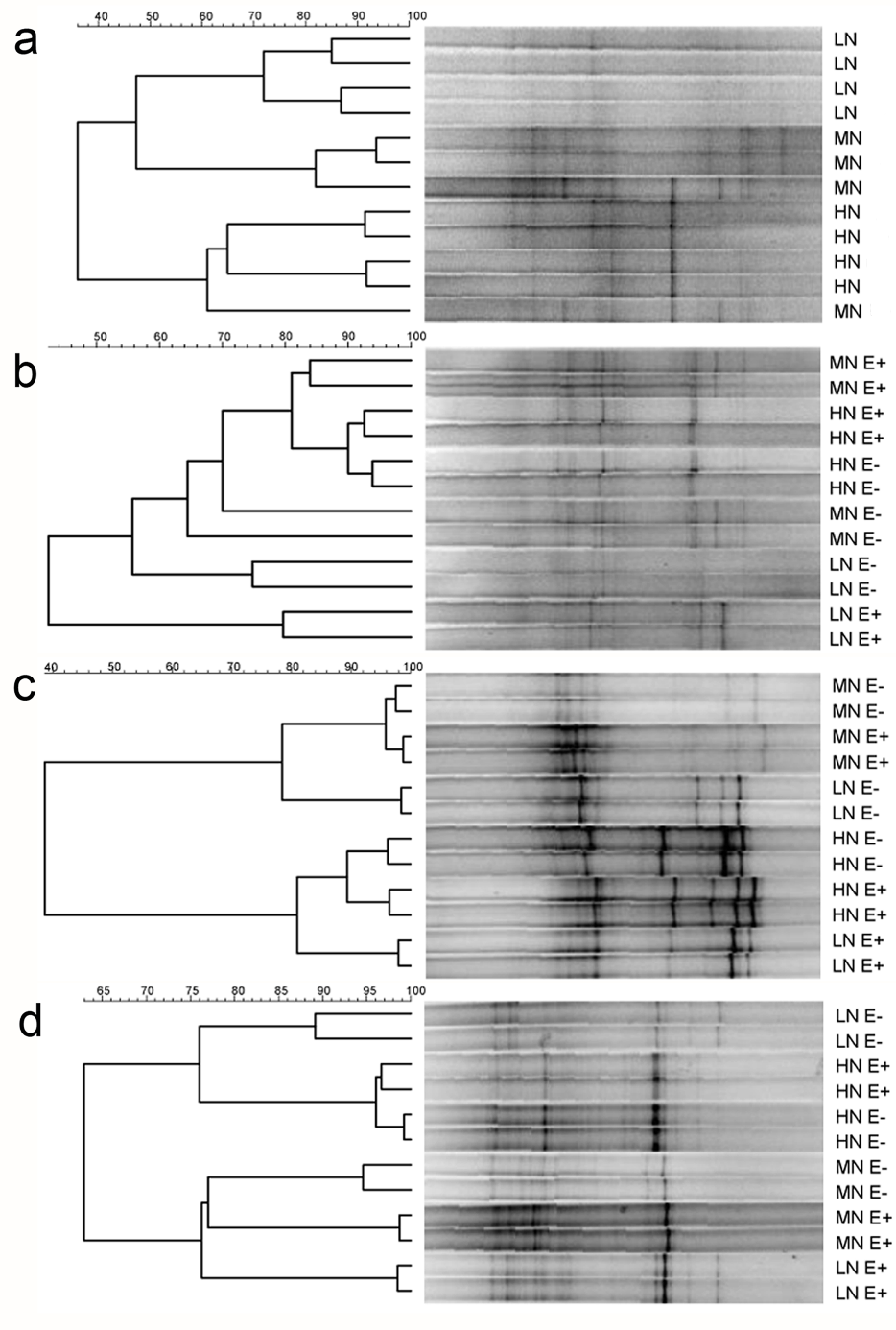


**Fig. S3** Denaturing gradient gel electrophoresis (DGGE) profile and analysis of soil ammonium-oxidizing bacteria communities. a-d: Cluster analysis of ammonium-oxidizing bacterial *amoA* genes from the DGGE pattern of four different stages of rice (a: S0, unplanted soil; b: S1, tillering; c: S2, grainfilling; d: S3, ripening). E+, endophyte infected; E-, endophyte uninfected; LN, low N; MN, medium N; HN, high N.


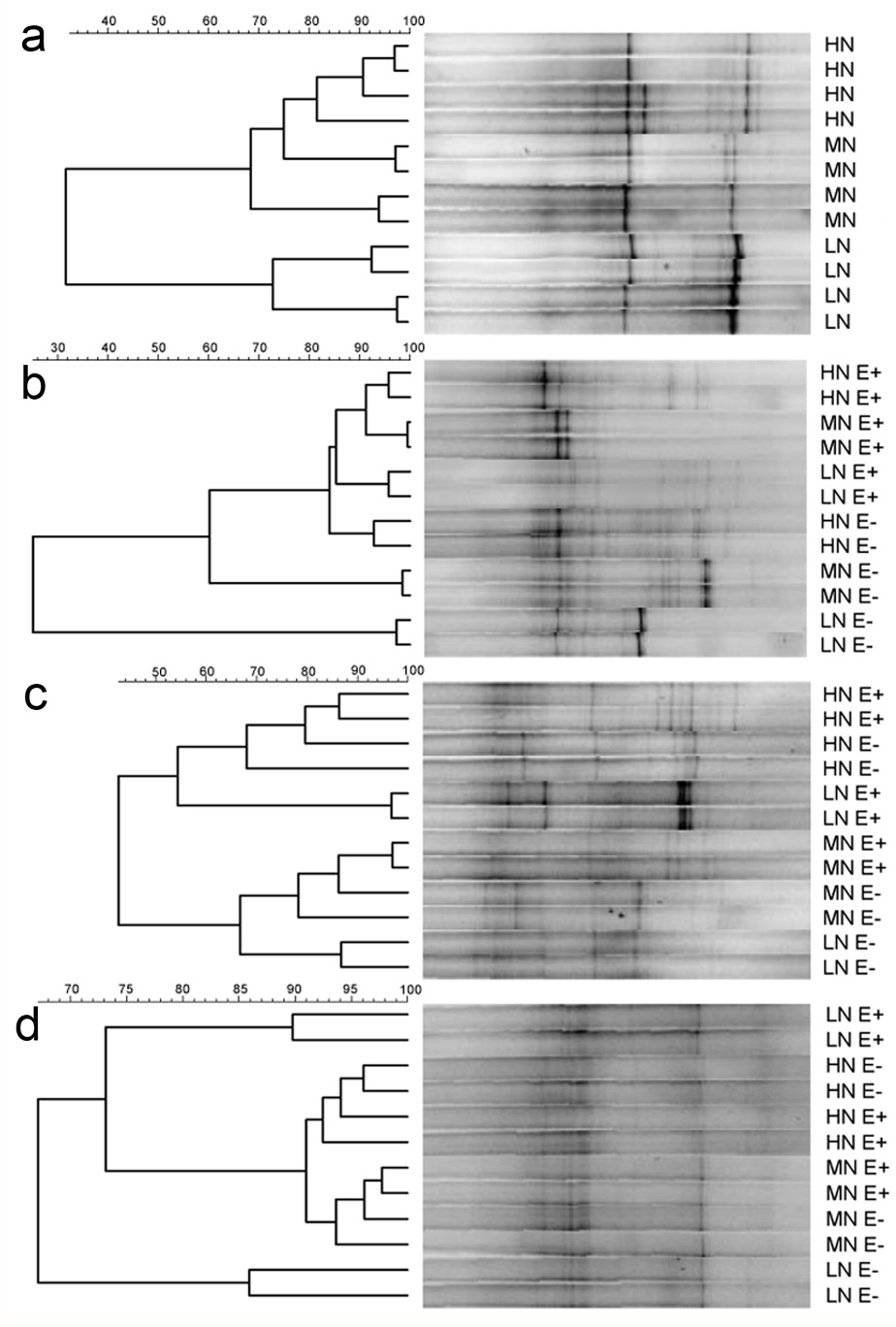


**Fig. S4** Denaturing gradient gel electrophoresis (DGGE) profile and analysis of soil diazotroph communities. a-d: Cluster analysis of diazotrophic *nifH* genes from the DGGE pattern of four different stages of rice (a: S0, unplanted soil; b: S1, tillering; c: S2, grainfilling; d: S3, ripening). E+, endophyte infected; E-, endophyte uninfected; LN, low N; MN, medium N; HN, high N.


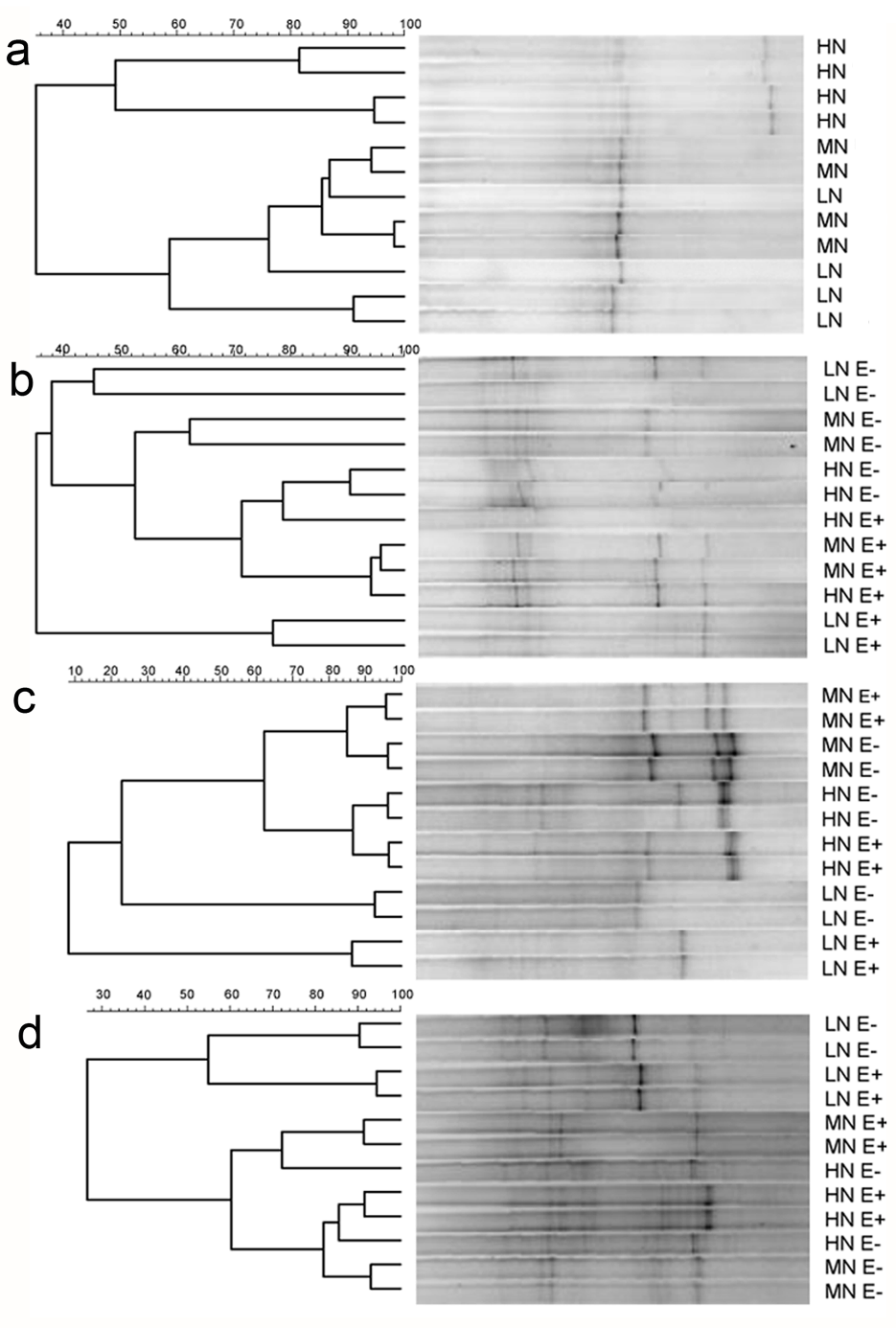


1. **Supplementary Tables**

**Table S1 Concentration of free amino acids in root exudates of different treatments**

| Free Amino acid concentration (μmol mL^-1^) | LN | | MN | | HN | |
| --- | --- | --- | --- | --- | --- | --- |
|  | E- | E+ | E- | E+ | E- | E+ |
| Asp | 1.54±0.22 | 4.24±0.37^**^ | 4.41±0.35 | 12.37±0.87^**^ | 7.41±0.45 | 6.67±0.59^*^ |
| Glu | 9.69±0.47 | 20.33±1.42^**^ | 22.37±1.09 | 26.88±1.54^**^ | 20.67±1.39 | 17.54±1.28 |
| Ser | — | — | — | — | 6.59±0.35 | 7.13±0.44 |
| Asn | 3.97±0.25 | 5.21±0.39^**^ | 5.12±0.33 | 13.51±0.67^**^ | 9.29±0.58 | 11.96±0.67^*^ |
| Gly | 4.38±0.42 | 3.69±0.67 | 3.38±0.90 | 7.00±1.09^**^ | 14.69±0.94 | 16.74±0.41^*^ |
| His | — | — | — | — | — | — |
| Gln | 19.84±0.78 | 23.99±1.03^**^ | 25.54±1.21 | 23.31±1.00 | 42.05±1.73 | 45.67±1.95 |
| GABA | 4.23±0.30 | 6.15±0.46^*^ | 4.69±0.53 | 4.79±0.58 | 4.09±0.45 | 3.36±0.52 |
| Thr | — | 6.27±0.23^**^ | — | 10.01±0.49^**^ | 24.51±0.65 | 22.54±0.75 |
| Ala | — | 30.19±0.69^**^ | 12.26±0.47 | 21.31±0.61^**^ | 32.26±0.57 | 44.56±0.73^**^ |
| Arg | — | — | — | — | — | 5.88±0.47^**^ |
| Pro | — | — | — | — | — | — |
| Tyr | 2.01±0.30 | 4.00±0.59^**^ | 3.61±0.41 | 4.21±0.57 | 14.61±0.47 | 15.31±0.63 |
| Val | 8.54±0.75 | 5.33±0.60^**^ | 4.59±0.61 | 5.46±0.74 | 16.39±0.68 | 15.28±0.54 |
| Met | — | — | — | — | — | 7.035±0.73^**^ |
| Cys | 5.40±0.37 | 5.82±0.28 | 8.98±0.62 | 5.31±0.54^**^ | 25.08±0.60 | 23.79±0.41 |
| Ile | 5.00±0.68 | 2.43±0.40^**^ | 3.29±0.44 | 2.89±0.32 | 12.83±0.47 | 13.99±0.62 |
| Leu | 2.13±0.37 | 2.11±0.25 | 2.70±0.34 | 2.56±0.28^**^ | 12.70±0.54 | 13.86±0.61 |
| Phe | — | — | — | 2.96±0.32^**^ | 10.77±0.98 | 12.40±1.08 |
| Trp | — | — | — | 1.24±0.09^**^ | — | 8.60±1.36^**^ |
| Oan | — | — | — | — | — | — |
| Lys | — | — | — | 5.09±0.51^**^ | 5.00±0.34 | 6.07±0.78 |
| Total | 66.72±12.08 | 119.74±14.17^**^ | 100.94±20.89 | 148.90±23.56^*^ | 267.94±24.84 | 298.38±26.05 |

Data are means ± SE from three biological replicates. * and ** indicate statistically significant differences between infected (E+) and uninfected (E-) plants (* *P* < 0.05; ** *P* < 0.01). LN, low N; MN, medium N; HN, high N.

**Table S2 Concentration of organic acids in root exudates of different treatments**

| Organic acid concentration  (μg mL^-1^) | LN | | MN | | HN | |
| --- | --- | --- | --- | --- | --- | --- |
|  | E- | E+ | E- | E+ | E- | E+ |
| Oxalic | — | 156.39±10.17^**^ | 264.13±18.56 | 302.47±21.33 | 463.23±26.73 | 501.53±30.17 |
| Tartaric | — | — | 397.94±38.25 | 461.15±52.61 | 309.64±23.25 | 379.16±42.55^**^ |
| Formic | — | — | — | — | — | — |
| Malic | — | 156.60±8.63^**^ | 84.85±5.64 | 237.06±14.47^**^ | 255.63±15.19 | 260.26±18.67 |
| α-Ketone | 11.31±0.49 | 67.47±3.81^**^ | 20.65±2.01 | 91.28±6.34^**^ | 87.27±4.47 | 232.94±13.76^**^ |
| Lactic | 105.43±6.73 | —^**^ | — | — | — | — |
| Acetic | 238.01±12.69 | 865.63±45.75^**^ | 612.08±24.56 | 706.59±36.95^*^ | 608.91±32.57 | 803.84±29.70^**^ |
| Citric | — | — | — | — | — | — |
| Fumaric | — | — | — | 1.96±0.12^**^ | — | — |
| Maleic | 2.14±0.06 | 2.20±0.11 | 2.13±0.04 | 2.19±0.09 | 0.97±0.02 | 4.542±0.19^**^ |
| Succinic | 543.41±23.27 | 231.72±14.08^**^ | 268.89±28.07 | 238.19±21.22 | 508.62±34.15 | 350.46±44.06^**^ |
| Total | 900.29±67.76 | 1479.40±109.31^**^ | 1650.77±187.38 | 2040.80±203.17 | 2234.26±199.25 | 2532.04±219.84 |

Data are means ± SE from three biological replicates. * and ** indicate statistically significant differences between infected (E+) and uninfected (E-) plants (* *P* < 0.05; ** *P* < 0.01). LN, low N; MN, medium N; HN, high N.

**Table S3** Two-way ANOVA for N-nutrient concentrations in the rhizospheric soil of *Phomopsis liquidambari-*infected (E+) and uninfected (E-) rice under different nitrogen (N) levels

|  |  | DF | Total N | | NH_4_^+^ | | NO_3_^-^ | | [NO_3_^-^/(NH_4_^+^ + NO_3_^-^)] (%) | |
| --- | --- | --- | --- | --- | --- | --- | --- | --- | --- | --- |
|  |  |  | F | P | F | P | F | P | F | P |
| S0 | N | 2 | 36.793 | <0.001 | 613.229 | <0.001 | 5510.243 | <0.001 | 1039.886 | <0.001 |
| S1 | E | 1 | 3.341 | 0.093 | 32.137 | <0.001 | 38.281 | <0.001 | 7.860 | 0.016 |
|  | N | 2 | 37.877 | <0.001 | 383.910 | <0.001 | 976.55 | <0.001 | 51.378 | <0.001 |
|  | E×N | 2 | 1.472 | 0.268 | 2.614 | 0.114 | 21.523 | <0.001 | 2.924 | 0.092 |
| S2 | E | 1 | 1.476 | 0.248 | 20.200 | <0.001 | 22.858 | <0.001 | 1.824 | 0.202 |
|  | N | 2 | 68.442 | <0.001 | 259.197 | <0.001 | 231.362 | <0.001 | 48.696 | <0.001 |
|  | E×N | 2 | 0.223 | 0.804 | 2.868 | 0.096 | 3.192 | 0.077 | 4.028 | 0.046 |
| S3 | E | 1 | 1.879 | 0.195 | 5.387 | 0.039 | 3.067 | 0.105 | 1.854 | 0.198 |
|  | N | 2 | 35.486 | <0.001 | 523.12 | <0.001 | 259.128 | <0.001 | 31.008 | <0.001 |
|  | E×N | 2 | 0.971 | 0.407 | 0.713 | 0.510 | 0.909 | 0.429 | 2.465 | 0.127 |

Data are means ± SE from three biological replicates. S0, unplanted soil; S1, tillering; S2, grainfilling; S3, ripening; LN, low N; MN, medium N; HN, high N. E, endophyte; N, nitrogen.

**Table S4** Two-way ANOVA for potential nitrification rates and N-transformation related gene copies in the rhizospheric soil of *Phomopsis liquidambari-*infected (E+) and uninfected (E-) rice under different nitrogen (N) levels

|  |  | DF | Potential nitrification rates | | *nifH* copies | | *amoA* copies | | *Arch-amoA* copies | |
| --- | --- | --- | --- | --- | --- | --- | --- | --- | --- | --- |
|  |  |  | F | P | F | P | F | P | F | P |
| S0 | N | 2 | 111.631 | <0.001 | 734.726 | <0.001 | 294.281 | <0.001 | 725.124 | <0.001 |
| S1 | E | 1 | 34.884 | <0.001 | 70.364 | <0.001 | 13.186 | 0.003 | 12.343 | 0.004 |
|  | N | 2 | 105.721 | <0.001 | 1469.125 | <0.001 | 190.160 | <0.001 | 148.722 | <0.001 |
|  | E×N | 2 | 1.920 | 0.189 | 11.809 | 0.001 | 10.770 | 0.002 | 7.641 | 0.007 |
| S2 | E | 1 | 2.641 | 0.130 | 2.542 | 0.137 | 14.369 | 0.003 | 6.632 | 0.024 |
|  | N | 2 | 50.011 | <0.001 | 889.114 | <0.001 | 408.716 | <0.001 | 72.666 | <0.001 |
|  | E×N | 2 | 6.804 | 0.011 | 3.871 | 0.050 | 6.879 | 0.010 | 0.521 | 0.607 |
| S3 | E | 1 | 0.226 | 0.643 | 0.098 | 0.760 | 0.678 | 0.426 | 0.350 | 0.565 |
|  | N | 2 | 24.253 | <0.001 | 240.208 | <0.001 | 121.071 | <0.001 | 40.216 | <0.001 |
|  | E×N | 2 | 0.092 | 0.913 | 0.140 | 0.870 | 0.374 | 0.696 | 0.419 | 0.667 |

Data are means ± SE from three biological replicates. S0, unplanted soil; S1, tillering; S2, grainfilling; S3, ripening; LN, low N; MN, medium N; HN, high N. E, endophyte; N, nitrogen.
